# Challenges and opportunities for converting renal cell carcinoma into a chronic disease with targeted therapies

**DOI:** 10.1038/sj.bjc.6606084

**Published:** 2011-02-01

**Authors:** M E Gore, J M G Larkin

**Affiliations:** 1 Department of Medicine, Royal Marsden Hospital, Fulham Road, London SW3 6JJ, UK

**Keywords:** chronic disease, combinatorial therapy, renal cell carcinoma, sequential therapy, sunitinib malate, targeted agents

## Abstract

Optimum efficacy is the primary goal for any cancer therapy, and entails controlling tumour growth and prolonging survival as far as possible. The prognosis for patients with metastatic renal cell carcinoma (mRCC) has greatly improved with the introduction of targeted therapies. This review examines the development and efficacy of targeted agents for the management of mRCC, the challenges offered by their rapid emergence, and discusses how mRCC treatment may evolve in the future. Improvements in progression-free survival and overall survival rates, observed with targeted agents, indicate that it may now be possible to change mRCC from a rapidly fatal and largely untreatable condition into a chronic disease. The major challenges to further advances in targeted therapy for mRCC include overcoming drug resistance, identifying the most effective sequence or combination of targeted agents, optimising clinical trial design and managing the cost of treatment.

Renal cell carcinoma (RCC) accounts for almost 2% of all adult malignancies (Parkin *et al*, 2005). There are 210 000 new cases diagnosed in the world each year, with more than 63 000 of these being in Europe (Parkin *et al*, 2005; Ferlay *et al*, 2007). Worldwide and in Europe, the annual number of deaths from this disease are 100 000 and 26 000, respectively (Ferlay *et al*, 2007). At diagnosis, approximately 20–30% of patients have metastatic renal cell carcinoma (mRCC) (Motzer *et al*, 1996), and a similar percentage of those with initially localised disease will subsequently relapse and develop metastases (Antonelli *et al*, 2007).

Historically, cytokine therapy was the only systemic treatment that had any consistent activity in mRCC. However, whereas cytokine treatment, particularly high-dose interleukin 2, can be associated with complete and durable responses, these occur in only a small proportion of patients (Fisher *et al*, 2000). In addition, cytokine treatment can be associated with substantial toxicity. In a Cochrane review of interferon-*α* (IFN-*α*) for mRCC, median overall survival (OS) for patients treated with this cytokine was found to be 11.4 months, which represented a 3.8-month improvement when compared with patients who received no immunotherapy (Coppin *et al*, 2004). In a randomised phase III study, a median OS of 17.5 months was observed in patients with mRCC, receiving high dose interleukin-2 (IL-2), compared with 13 months, for those treated with a combination of lower dose of IL-2 and IFN-*α*, but this difference was not statistically significant (*P*=0.211) (McDermott *et al*, 2005). In addition, it has become clear that only patients with good prognostic features are likely to benefit from immunotherapy. This is further supported by the recent results of McDermott *et al* (2010) who demonstrated a higher response rate in patients, who were selected based on clinical and pathological features, treated with high-dose IL-2 when compared with historical data (28 *vs* 14% *P*=0.0016).

The development of targeted agents for the treatment of mRCC has provided physicians with unprecedented opportunities to improve clinical outcomes for patients with mRCC. For example, compared with IFN-*α*, treatment, the oral multi-targeted receptor tyrosine kinase (RTK) inhibitor, sunitinib malate (Sutent; Pfizer Inc., New York, NY, USA) doubled median progression-free survival (PFS) in a phase III trial of patients with previously untreated mRCC (Motzer *et al*, 2007). In addition, sunitinib was associated with median OS of greater than 2 years in this trial and substantial improvements in objective response rates when compared with IFN-*α* treatment (Motzer *et al*, 2007, 2009).

Optimum efficacy is the primary goal of any cancer treatment and this means controlling tumour growth as far as possible and prolonging survival. The new-targeted agents such as sunitinib, with their improved response and survival rates, have allowed this goal to be realisable for patients with mRCC. We now have the therapeutic tools that could potentially result in mRCC changing from a rapidly fatal, largely untreatable condition, into a chronic disease. Several treatment challenges must be overcome by us to maximise the potential of targeted agents, and these include the identification of predictive molecular markers, drug resistance, the identification of the most effective sequence or combination of targeted agents, efficient clinical trial design and the provision of cost-effective access to treatment for all patients with mRCC.

This review examines the development and efficacy of targeted agents for the management of mRCC, discusses the challenges offered by their rapid emergence and speculates how mRCC treatment might evolve in the future.

## Development and efficacy of targeted agents

Advances in our understanding of the molecular mechanisms associated with RCC have enabled rational targets for systemic therapy to be identified. In clear cell RCC, inactivation of the *von Hippel-Lindau* gene is associated with the accumulation of both hypoxia-inducible factor 1 (HIF-1*α*) and HIF-2*α*. HIF-1*α* causes transcriptional activation of several genes, including vascular endothelial growth factor (*VEGF*) and platelet-derived growth factor (*PDGF*), both of which are implicated in tumour angiogenesis and growth (Krause and Van Etten, 2005). These ligands serve as agonists for their respective RTKs, VEGF receptor (*VEGFR*) and *PDGF* receptor (PDFGR). HIF-1*α* activity is also regulated by other growth factor and cell adhesion pathways (Motzer and Bukowski, 2006); for example, HIF-1*α* is increased in response to growth factor binding to the phosphatidylinositol 3-kinase/Akt/mammalian target of rapamycin (mTOR) and Ras/Raf/mitogen-activated protein kinase signalling pathways. In contrast, HIF2-*α* has an opposing role to HIF-1*α* with regard to gene expression, and promotes enhanced tumour growth (Toschi *et al*, 2008; Biswas *et al*, 2010). Elevation of HIF2-*α* expression also contributes towards angiogenesis (Toschi *et al*, 2008).

The formation of a multi-molecular complex between mTOR and regulatory-associated protein of mTOR (raptor) has a role in cell proliferation, survival and tumour angiogenesis (Le Tourneau *et al*, 2008). Upregulation of VEGF or PDGF can also stimulate activity of mTORC1 (Le Tourneau *et al*, 2008). In addition, mTOR is also able to form a complex with rapamycin-insensitive companion of mTOR (rictor) to form mTORC2 (Le Tourneau *et al*, 2008). Development of targeted therapies has focused on mTORC1 and other components of the HIF-1*α* signalling pathway, aimed at reducing the effects of growth factors, including VEGF and PDGF.

Sunitinib is a multi-targeted RTK inhibitor, targeting a range of receptors including VEGFR-1, -2 and -3, PDGFR-*α* and -*β*, glial cell line-derived neurotrophic factor receptor (REarranged during Transfection; RET), the receptor for macrophage colony-stimulating factor 1, FMS-like tyrosine kinase 3 receptor (FLT3) and c-KIT (Motzer *et al*, 2007). Sorafenib (Nexavar; Bayer Healthcare, Leverkusen, Germany) and pazopanib (Votrient; GlaxoSmithKline, Middlesex, UK), are also both oral multi-targeted RTK inhibitors, with sorafenib targeting VEGFR-2 and -3, PDGFR-*β*, FLT3, c-KIT, RET, B-Raf and Raf-1/C-Raf (Escudier *et al*, 2009a) and pazopanib targeting VEGFR-1, -2 and -3, PDGFR-*α* and -*β* and c-KIT (Sternberg *et al*, 2010a). Temsirolimus (Torisel; Pfizer Inc) and everolimus (Afinitor; Novartis, Basel, Switzerland) are both mTOR kinase inhibitors (Hudes *et al*, 2007; Motzer *et al*, 2008) and bevacizumab (Avastin; F Hoffmann-La Roche, Basel, Switzerland) is a humanised monoclonal antibody that binds to and neutralises all major isoforms of VEGF-A (Escudier *et al*, 2010a).

Sunitinib is approved multinationally for the treatment of mRCC, and is a reference standard of care for the first-line treatment of patients with mRCC (Escudier *et al*, 2010b). Other targeted agents that are approved in the first-line setting include temsirolimus for the treatment of mRCC patients, with poor prognostic factors, and the combination of bevacizumab plus IFN-*α*. Sorafenib is approved for patients with mRCC who have failed, or are considered unsuitable for, cytokine therapy. Everolimus is approved for the treatment of patients with mRCC following the failure of treatment with sunitinib or sorafenib (Escudier *et al*, 2010b). Most recently, pazopanib has been approved in the USA for the treatment of advanced RCC, and received conditional approval in Europe for the treatment of advanced RCC in the first-line setting and in patients with previous cytokine therapy. In contrast to sunitinib, sorafenib and pazopanib, temsirolimus and bevacizumab both have single targets and are administered intravenously. Everolimus also has a single target, but is orally administered.

Additional targeted agents are currently under investigation for mRCC. Those in phase III of development include axitinib, an orally administered multi-targeted receptor RTK inhibitor (Rini *et al*, 2007; Rixe *et al*, 2007) and tivozanib, an oral VEGFR-targeted tyrosine kinase inhibitor (Bhargava *et al*, 2009). Both these agents are similar in terms of mechanisms of action to the currently available VEGF-targeted therapies, and differ from these agents on the basis of affinities for the various receptors (Eskens *et al*, 2008; Schmidinger and Bellmunt, 2010). The clinical efficacy demonstrated by the targeted agents approved for the treatment of mRCC, and the most promising of those currently in development, are summarised in Tables 1, 2 and 3.

## Targeted agents and drug resistance

Targeted agents have significantly improved the prognosis for patients with mRCC; however, complete responses are rare and the majority of patients develop drug resistance, as exemplified by the fact that their disease progresses during treatment (Sosman *et al*, 2007). Drug resistance is the underlying reason for the growth and spread of tumours in the presence of systemic treatment, and it is the main barrier against long-term tumour control.

**Figure 1:**
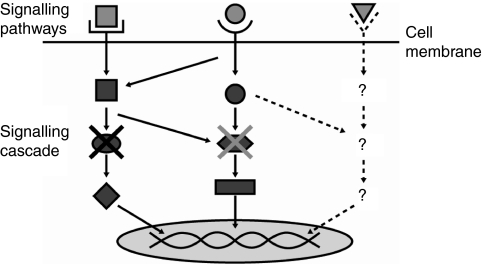
Resistance to targeted agents may occur through target bypass mechanisms. A targeted agent (denoted by X in the figure) may effectively inhibit the signalling cascade of one or more pathway (denoted by the light grey square and circle in the figure). However, the presence of a ‘bypass’ mechanism may allow this inhibition to be circumvented by signalling along an unknown or unrelated pathway (denoted by the light grey triangle in the figure), resulting in resistance to the targeted therapy.

Resistance to targeted agents in mRCC may develop as a result of the multiplicity of pathways involved in regulating HIF-1*α* activity, some of which have not yet been identified. A particular targeted agent may effectively inhibit one or more pathways, but resistance can result from the development of other molecular and cellular processes that ‘bypass’ this effect (Figure 1). These include additional mutations, feedback loops compensating for inhibition through gene upregulation, increased angiogenesis or the activation of downstream mediators (Sosman *et al*, 2007). Furthermore, some of these bypass mechanisms may not even involve the VEGF pathway. For example, a preclinical study demonstrated that upregulation of proangiogenic pathways, through increased expression of IL-8, contributed towards evasion of anti-angiogenic effects mediated by sunitinib (Huang *et al*, 2010).

Rini and Flaherty (2008) have described three clinical patterns of resistance in patients with mRCC: a small group (15–20%), which is resistant to therapy from the outset of treatment, a larger group that demonstrates early-tumour regression followed by a short period of stability then disease progression (6–12 months from the start of therapy) and finally a subgroup of patients that exhibit tumour response over several months followed by a prolonged period of stable disease, without the appearance of new lesions.

Additional reasons for the development of resistance to treatment may include pharmacokinetic resistance to treatment whereby the pharmacokinetic activity of the treatment may be affected by interaction with cellular proteins or structure such as transport pumps (Schmidt, 2008). Non-compliance with oral treatment may also result in the development of resistance to treatment (Schmidt, 2008).

The mode of action of targeted agents will affect the strategies that will be utilised to overcome drug resistance. Multi-targeted agents may provide the most effective option for combating drug resistance because of involvement of multiple signalling pathways in the pathology of RCC. Differential responses to targeted agents have been observed between tumours within the same patient, and also between tumours within the same organ. This indicates variances in resistance between tumour clones and the use of multi-targeted agents, such as sunitinib or sorafenib, instead of single-targeted agents, might be most effective at simultaneously blocking, both known and unknown, angiogenic and proliferation pathways.

A major consideration in devising the optimal treatment strategy to overcome drug resistance, and thus optimising long-term therapy with targeted agents in mRCC is how best to use these agents in sequence or in combination. Data in favour of either sequencing or combining targeted agents for mRCC are currently limited, and as discussed below, clinical studies are ongoing to investigate both these approaches.

## Sequential therapy with targeted agents

Targeting different pathways through sequential therapy should offer benefit in terms of overcoming resistance to individual agents. It also enables a treatment continuum to be achieved, maintaining patients on treatment without progression for as long as possible. Sequential therapy has the potential to change mRCC into a chronic disease that can be managed for long term through the administration of targeted agents in sequence. It should also enable full dosages of targeted agents to be administered, ensuring that optimal drug levels are achieved without the additional toxicity that often occurs with combinatorial approaches. There is emerging evidence that dose is important with the targeted agents in this disease. The results of a meta-analysis of data from sunitinib studies indicated that higher sunitinib exposure is associated with higher efficacy with respect to longer times to tumour progression and OS (Houk *et al*, 2010). In practice, clinicians are currently using targeted agents in a sequential manner for patients with mRCC, although concerns remain regarding cross-resistance between the different agents, and there are many questions regarding the optimal sequence for obtaining maximal clinical benefit from the available targeted therapies.

Current data indicate that there is a degree of non-cross-resistance between the different targeted agents. A retrospective study by Tamaskar *et al* (2006) found that both sunitinib and sorafenib demonstrated anti-tumour activity in patients refractory to previous anti-angiogenic therapy. In particular, clinical benefit was observed in patients receiving sorafenib following previous therapy with sunitinib and *vice versa*. Similarly, a lack of cross-resistance between sunitinib and sorafenib was observed in another retrospective analysis of 90 patients, supporting sequential use of these agents in the treatment of mRCC (Sablin *et al*, 2007). The results of a randomised phase II study of sorafenib alone and in combination with low-dose IFN-*α* following previous first-line sunitinib treatment in patients with mRCC are awaited (CONCERT study) (http://www.clinicaltrials.gov).

Incomplete cross-resistance has also been demonstrated between sunitinib and bevacizumab. Rini *et al* (2008) treated 61 bevacizumab-refractory patients with sunitinib; an objective response rate of 23% (95% CI: 13.2–35.5) and median PFS of 30.4 weeks (95% CI: 18.3–36.7) were achieved. All these data suggest that resistance to one VEGF-targeted therapy can be overcome by another agent that also targets this pathway.

Interestingly, transient resistance to the same agent has also been observed. In a recent retrospective review of 23 patients, re-challenge with sunitinib in patients with disease progression on sunitinib and other therapies, resulted in 5 patients (22%) achieving PR and 17 patients (74%) achieving SD (Rini *et al*, 2010a). Re-challenge was associated with a median PFS of 7.2 months compared with 13.7 months on initial treatment (*P*=0.04). In addition, patients with more than 6 months between sunitinib treatments had significantly longer PFS than those receiving re-treatment with sunitinib within 6 months (16.5 and 6.0 months, respectively). The results described here indicate the potential for re-treating with an agent despite the occurrence of resistance at first treatment and have implications for achieving a continuum of treatment in these patients.

The first randomised phase III study to investigate sequential targeted therapy in mRCC showed clinical efficacy for the sequence of sunitinib or sorafenib, followed by everolimus (RECORD-1) (Escudier *et al*, 2008; Motzer *et al*, 2008, 2010). In this study, patients who had failed earlier anti-VEGF therapy, 71% of whom had received sunitinib previously, were treated with either everolimus or placebo. The median PFS was 4.9 *vs* 1.9 months for those treated with everolimus or placebo, respectively (*P*<0.001; hazard ratio (HR) 0.33 (95% CI: 0.25–0.43)). Improvements in PFS with everolimus relative to placebo were observed across all Memorial Sloan–Kettering Cancer Center (MSKCC) prognostic risk groups. Patients pre-treated with sunitinib achieved a median PFS of 3.9 *vs* 1.8 months when treated with everolimus or placebo, respectively (*P*<0.001; HR 0.34 (95% CI: 0.23–0.51)). Everolimus-treated sorafenib-refractory patients achieved a median PFS of 5.9 *vs* 2.8 months for those treated with placebo (*P*<0.001; HR 0.25 (95% CI: 0.16–0.42)). Patients refractory to both sunitinib and sorafenib achieved a median PFS of 4.0 months when treated with everolimus compared with 1.8 months for those treated with placebo (*P*<0.001; HR 0.32 (95% CI: 0.19–0.54)).

Sequential treatment has also been investigated with the VEGF inhibitor, axitinib. In a phase II study, median PFS was 7.1, 9.0, and 7.7 months for those mRCC patients who had received previous treatment with sunitinib and sorafenib, cytokines and sorafenib, or sorafenib alone, respectively (Dutcher *et al*, 2008). The phase III AXIS study investigates axitinib in 540 patients with mRCC who have experienced failure on a first-line treatment, including sunitinib, bevacizumab plus IFN-*α*, temsirolimus or cytokines (http://www.clinicaltrials.gov). A phase III study comparing sorafenib with temsirolimus (INTORSECT study) will also investigate sequential therapy in an estimated 440 patients who have failed first-line sunitinib (Bhojani *et al*, 2008).

**Figure 2:**
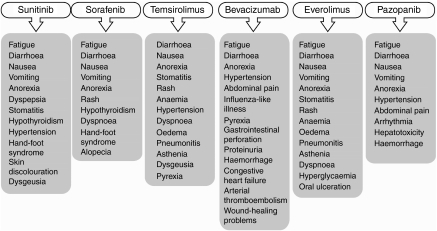
Most common and side effects of interest reported with the six licensed targeted agents for mRCC.

In the context of sequential therapy, adequate management of treatment-related toxicity can allow patients to remain on treatment for long periods and help maximise the clinical benefit of targeted agents. The toxicity profile of each of the targeted agents approved for the treatment of mRCC is well defined (Figure 2), and strategies to manage treatment-related adverse events are being refined (Bhojani *et al*, 2008). In addition, clinicians’ familiarity with targeted agents is increasing and this experience is accompanied by the ability to manage treatment-related adverse events more effectively. Effective therapy management involves optimisation of dose, maximising treatment duration and a proactive approach to the management of toxicities.

## Combination therapy with targeted agents

Combining therapeutic agents may also overcome drug resistance and allow for the simultaneous inhibition of multiple signalling pathways. These combinations could consist of agents blocking a single target, single and/or multi-targeted agents, and targeted agents combined with cytotoxics, cytokines or other therapeutic agents. This approach presupposes knowledge of all the signalling pathways involved in the development and continuing growth of a tumour. Furthermore, any potential clinical benefit must be balanced against the potential increase in toxicity associated with combining therapeutic agents.

Combination therapies are currently under investigation in several ongoing and planned clinical trials. These include several studies evaluating bevacizumab in combination with temsirolimus, everolimus and sorafenib (Merchan *et al*, 2007; Sosman *et al*, 2008; Whorf *et al*, 2008). Two phase I studies have evaluated sunitinib in combination with bevacizumab for the treatment of solid tumours, including mRCC (Feldman *et al*, 2007; Rini *et al*, 2010c). Results from these studies have noted that the combination is associated with significant toxicity at full-doses of sunitinib and bevacizumab (Feldman *et al*, 2007; Rini *et al*, 2009a). Similarly, treatment of patients with mRCC using sorafenib in combination with bevacizumab does not seem to be possible at full doses of both drugs (Sosman *et al*, 2006). A further phase I study, in which temsirolimus was combined with sunitinib, was terminated because of dose-limiting toxicity observed at low-starting doses of both agents (Patel *et al*, 2009). Sorafenib plus IFN-*α*2b demonstrated clinical activity for the first-line treatment of patients with mRCC, but the toxicity profile of this combination has limited its development in relation to the use of full doses of both these agents within such a combination (Ryan *et al*, 2007). Another study, evaluating the efficacy of bevacizumab combined with everolimus demonstrated clinical activity of the combination for the treatment of patients with advanced RCC, although the efficacy results did not demonstrate a clear advantage for the combination over single agent, sequential treatment and the occurrence of grades 3–4 proteinuria was higher than expected in this study (Hainsworth *et al*, 2010). The results of these early studies demonstrate the potential for detrimental effects for the combination of therapeutic agents. Further larger studies are required to determine the clinical applicability if any, of other targeted agent combinations.

**Figure 3:**
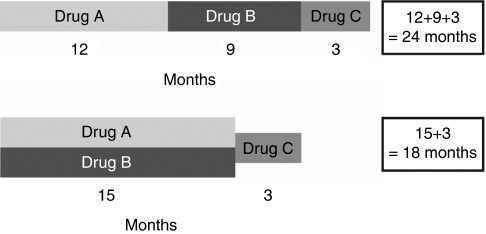
Treatment strategies with targeted agents involve a sequential or a combinatorial approach; single-agent sequential therapy may cause tumour shrinkage but may also slow disease progression and, therefore, turn mRCC into a chronic disease.

It is also important to consider the therapeutic options that are possible or available following the use of combination-targeted agent therapy. The development of resistance to combination-targeted therapy could negatively impact subsequent treatment. This theoretical consideration relates to the possibility that combination therapy will ‘use’ up active treatment options, and denies the potential for the repeated ‘beneficial’ interventions that the single-agent sequential therapy strategy affords (Figure 3).

## Targeted agents and clinical trial design

The approval of sunitinib, sorafenib, temsirolimus, everolimus, bevacizumab plus IFN-*α* and pazopanib in Europe for the treatment of mRCC, resulted in the establishment of evidence-based recommendations and a treatment algorithm that can be used to achieve optimal clinical benefit with these agents. Data from randomised phase III clinical trials were, of course, an essential element in this process (Escudier *et al*, 2010b).

A large number of new targeted therapeutic compounds are currently under investigation for mRCC. Using traditional ‘pick the winner’ trials to demonstrate the efficacy of these agents in mRCC could result in the failure of some compounds, as current trial designs and statistical methods may not be sensitive enough to evaluate their therapeutic benefits, particularly as they relate to subgroups of mRCC patients, for example, those with comorbidities, histological and molecular subtypes.

In addition, the end points used in phase II and III clinical trials might not always be appropriate for assessing the efficacy of new agents (Medina *et al*, 2007). Standard cytotoxic agents rely on response rates, time to tumour progression, PFS and OS as clinical end points to assess efficacy. Targeted therapies may have both a cytotoxic and a cytostatic effect, complicating assessment of response to treatment. As such, clinical benefit consisting of a partial response and/or disease stabilisation, that is, ‘slowing’ of disease progression could be a clinically relevant new parameter of efficacy. It is this concept, together with the strategy of single-agent sequential therapy, which could transform mRCC into a chronic condition (Figure 3). The most appropriate end points for assessing efficacy of new targeted agents are currently under debate. Biomarkers serving as surrogates for efficacy, functional imaging technology and a focus on new clinical end points, are all required to help further develop this area of novel therapeutics (Medina *et al*, 2007; Sessa *et al*, 2008).

Biomarkers may identify patients with the potential to benefit from targeted therapy, and may be predictive of response, allowing a more individualised approach to prognostication and treatment. The expression of several molecular markers, including circulating VEGF, endostatin and cell surface markers, such as CD31, may all be relevant in this context (Sessa *et al*, 2008). These biomarkers may be useful in predicting treatment outcomes in mRCC, but they have not yet been validated for use in routine clinical practice. Imaging studies can also contribute towards determining the efficacy of an agent under development, providing useful information regarding early-treatment response by monitoring alterations in tumour vasculature and angiogenesis (Sessa *et al*, 2008). However, the implementation of multiple assessments for each patient could prove to be prohibitively costly (Sessa *et al*, 2008).

## Access to targeted agents

Considerations of cost can impact the availability, timing and duration of treatment with targeted therapies. It may also influence the choice of treatment strategy used to overcome drug resistance and how response to therapy is assessed. Commissioners and providers of healthcare are increasingly looking to achieve the most cost-effective outcomes by balancing clinical efficacy against patient management costs, for example, costs of managing adverse events and drug pricing. Recently, the cost-effectiveness of sunitinib for the first-line treatment of patients with mRCC was evaluated by the National Institute for Health and Clinical Excellence (NICE) in the United Kingdom. The NICE Assessment Group developed an analysis model to evaluate the cost-effectiveness of sunitinib in comparison with IFN-*α* as well as the other therapies approved for the treatment of mRCC, and concluded that sunitinib provided a cost-effective option for the treatment of patients with mRCC in the first-line setting.

The NICE finding mirrors the economic analyses conducted using data from phase II and III trials of sunitinib, sorafenib, bevacizumab plus IFN-*α* and temsirolimus *vs* IFN-*α* as first-line therapy for patients with mRCC in the USA, Sweden and Spain (Oudard *et al*, 2010). On the basis of indirect treatment comparisons, these analyses identified sunitinib as a cost-effective alternative to sorafenib, bevacizumab plus IFN-*α* and temsirolimus in the first-line treatment of patients with mRCC. Cost-effectiveness ratios of sunitinib *vs* other therapies were within the established threshold that society is willing to pay for health benefits.

## Conclusion

The development of targeted agents has substantially improved the prognosis for patients with mRCC and has the potential to convert mRCC into a chronic disease.

Challenges in achieving this goal include:

- identifying and optimising the most appropriate sequence or combination of agents.
- development of molecular biomarkers to better identify the patients who are likely to benefit from a particular agent.
- imaging techniques as predictive markers of efficacy to minimise the time on treatment for those patients who will not respond to a particular agent.
- developing appropriate clinical trial designs and statistical methods to test new therapies.
- overcoming drug resistance by more new agents, or sequential or combination therapies.
- managing the cost barriers against treatment.

## Figures and Tables

**Table 1:** Clinical efficacy data for targeted agents approved in Europe/USA for the treatment of mRCC in the first-line setting

	**Number of patients (*N*)**	**Median PFS (months)**	***P*-value**	**Median OS (months)**	***P*-value**
Sunitinib (Motzer *et al*, 2009)	375	11	<0.001	26.4	0.051
*vs* IFN-*α*	360	5		21.8	0.049^a^
Temsirolimus^†^ (Hudes *et al*, 2007)	209	5.5	0.0001	10.9	0.0069
*vs* IFN-*α*	207	3.1		7.3	
Bevacizumab (plus IFN-*α*) (Escudier *et al*, 2010a)	327	10.2	<0.0001	23.3	0.1291
*vs* IFN-*α*	322	5.4		21.3	
Bevacizumab (plus IFN-*α*) (Rini *et al*, 2009b, 2010b)	369	8.5	<0.0001	18.3	0.069
*vs* IFN-*α*	363	5.2		17.4	
Sorafenib (Escudier *et al*, 2009b)	97	5.7	0.504	NR	NA
*vs* IFN-*α*	92	5.6			
Pazopanib (Sternberg *et al*, 2010a, 2010b) (overall)	290	9.2	<0.0001	22.9	0.224
*vs* placebo (overall)	145	4.2		20.5	
Treatment-naïve patients	155	11.1	<0.0001	NR	NA
*vs* placebo	78	2.8			

Abbreviations: IFN-*α*=interferon-alfa; NA=not applicable; NR=not reported; OS=overall survival; PFS=progression-free survival.

a *P*-values by pre-planned unstratified and stratified log-rank test, respectively.

^†^Patients stratified into the poor-risk prognostic category on the basis of three of six risk features (five pre-defined Memorial Sloan–Kettering Cancer Centre risk factors plus multiple sites of organ metastases).

**Table 2:** Clinical efficacy data for targeted agents approved in Europe/USA for the treatment of mRCC in the second-line setting

	**Number of patients (*N*)**	**Primary treatment**	**Median PFS (months)**	***P*-value**	**Median OS (months)**	***P*-value**
Sorafenib (Escudier *et al*, 2009a)	451	Systemic therapy with cytokines	5.5	<0.001	17.8	0.0287
*vs* placebo	452		2.8		14.3	
Everolimus (Escudier *et al*, 2008; Motzer *et al*, 2009, 2010) (overall)	272	Previous VEGF inhibitor therapy (sunitinib, sorafenib or both; bevacizumab permitted); systemic therapy with cytokines	4.9	<0.001	14.8	0.177
*vs* placebo (overall)	138		1.9		14.4	
refractory to sunitinib	124		3.9	<0.001	NR	NA
*vs* placebo	60		1.8			
refractory to sorafenib	77		5.9	<0.001	NR	NA
*vs* placebo	42		2.8			
Pazopanib (Sternberg *et al*, 2010a, 2010b) (overall)	290	Previous systemic therapy with cytokines	9.2	<0.0001	22.9	0.224
*vs* placebo (overall)	145		4.2		20.5	
cytokine-pre-treated patients	135		7.4	<0.001	NR	NA
*vs* placebo	67		4.2			

Abbreviations: NA=not applicable; NR=not reported; OS=overall survival; PFS=progression-free survival.

**Table 3:** Clinical efficacy data for targeted agents in development for the treatment of mRCC

	**Study design**	**Median PFS (months)**	***P*-value**	**Median OS (months)**	***P*-value**
Reyorafenib (Eisen *et al*, 2009)	First line, phase II	8.3	NA	NR	NA
Tivozanib (Bhargava *et al*, 2009)	First line, phase II	11.8	NA	NR	NA
Axitinib (Rixe *et al*, 2007)	Second line, phase II	15.7^a^	NA	29.9	NA
Axitinib (Rini *et al*, 2007; Dutcher *et al*, 2008)	Second line, phase II	7.4	NA	NR	NA
Refractory to sunitinib and sorafenib		7.1			
Refractory to cytokines and sorafenib		9			
Refractory to sorafenib		7.7			
Linifanib (Tannir *et al*, 2009)	Second line, phase II	5.4	NA	15.7	NA
Cediranib (Mulders *et al*, 2009)	First/second line, phase II	12.1	0.017	NR	NA
*vs* placebo		2.8			

Abbreviations: NA=not applicable; NR=not reported; OS=overall survival; PFS=progression-free survival.

a Reported as time to progression.

## References

[bib1] Antonelli A, Cozzoli A, Zani D, Zanotelli T, Nicolai M, Cunico SC, Simeone C (2007) The follow-up management of non-metastatic renal cell carcinoma: definition of a surveillance protocol. BJU Int 99: 296–300 17326263 10.1111/j.1464-410x.2006.06616.x

[bib2] Biswas S, Troy H, Leek R, Chung Y-L, Li J, Raval RR, Turley H, Gatter K, Pezzella F, Griffiths JR, Stubbs M, Harris AL (2010) Effects of HIF-1*α* and HIF2*α* on growth and metabolism of clear-cell renal cell carcinoma 786-0 xenografts. J Oncol 2010: 757908 20652061 10.1155/2010/757908 PMC2905950

[bib3] Bhargava P, Esteves B, Nosov DA, Lipatov ON, Anishchenko AA, Chacko RT (2009) Updated activity and safety results of a phase II randomized discontinuation trial (RDT) of AV-951, a potent and selective VEGFR1, 2, and 3 kinase inhibitor, in patients with renal cell carcinoma (RCC). J Clin Oncol 27: (Abstract 5032) 10.1200/JCO.2011.35.3524 22493422

[bib4] Bhojani N, Jeldres C, Patard JJ, Perrotte P, Suardi N, Hutterer G, Patenaude F, Oudard S, Karakiewicz PI (2008) Toxicities associated with the administration of sorafenib, sunitinib, and temsirolimus and their management in patients with metastatic renal cell carcinoma. Eur Urol 53: 917–930 18054825 10.1016/j.eururo.2007.11.037

[bib5] Coppin C, Porzsolt F, Autenrieth M, Kumpf J, Coldman A, Wilt T (2004) Immunotherapy for advanced renal cell cancer. Cochrane Database Syst Rev (3): CD001425 10.1002/14651858.CD001425 10908496

[bib6] Dutcher JP, Wild D, Hudes GR, Stadler WM, Kim S, Tarazi JC, Rosbrook B, Rini BI (2008) Sequential axitinib (AG-013736) therapy of patients (pts) with metastatic clear cell renal cell cancer (RCC) refractory to sunitinib and sorafenib, cytokines and sorafenib, or sorafenib alone. Poster presented at the 44th Annual Meeting of the American Society of Clinical Oncology, Chicago, Illinois, 30 May–3 June, 2008 (Abstract 5127)

[bib7] Eisen T, Joensuu H, Nathan P, Harper P, Wojtukiewicz M, Nicholson S, Bahl A, Tomczak P, Wagner A, Quinn D (2009) Phase II trial of the oral multikinase inhibitor BAY 73-4506 as 1st-line therapy in patients with metastatic or unresectable renal cell carcinoma (RCC). Eur J Cancer Supplements 7, Abstract 7.105; oral

[bib8] Escudier B, Bellmunt J, Négrier S, Bajetta E, Melichar B, Bracarda S, Ravaud A, Golding S, Jethwa S, Sneller V (2010a) Phase III trial of bevacizumab plus interferon alfa-2a in patients with metastatic renal cell carcinoma (AVOREN): final analysis of overall survival. J Clin Oncol 28: 2144–2150 20368553 10.1200/JCO.2009.26.7849

[bib9] Escudier B, Eisen T, Stadler WM, Szczylik C, Oudard S, Staehler M, Negrier S, Chevreau C, Desai AA, Rolland F, Demkow T, Hutson TE, Gore M, Anderson S, Hofilena G, Shan M, Pena C, Lathia C, Bukowski RM (2009a) Sorafenib for treatment of renal cell carcinoma: final efficacy and safety results of the phase III treatment approaches in renal cancer global evaluation trial. J Clin Oncol 27: 3312–3318 19451442 10.1200/JCO.2008.19.5511

[bib10] Escudier B, Kataja V (2010b) Renal cell carcinoma: ESMO clinical practice guidelines for diagnosis, treatment and follow-up. Ann Oncol 21(Suppl 5): v137–v139 20555064 10.1093/annonc/mdq206

[bib11] Escudier B, Ravaud A, Oudard S, Hutson TE, Porta C, Bracarda S, Grunwald V, Thompson J, Figlin RA, Motzer RJ (2008) Phase-3 randomised trial of everolimus (RAD001) vs placebo in metastatic renal cell carcinoma. Ann Oncol 19(Suppl. 8): viii45 (Abstract 72O)

[bib12] Escudier B, Szczylik C, Hutson TE, Demkow T, Staehler M, Rolland F, Negrier S, Laferriere N, Scheuring UJ, Cella D, Shah S, Bukowski RM (2009b) Randomized phase II trial of first-line treatment with sorafenib versus interferon Alfa-2a in patients with metastatic renal cell carcinoma. J Clin Oncol 27: 1280–1289 19171708 10.1200/JCO.2008.19.3342

[bib13] Eskens F, de Jonge M, Esteves B, Cotreau M, Bhargava P, Ryan J, van Doorn L, Isoe T, Hayashi K, Ekman L, Burger H, Verweij J (2008) Updated results from a Phase I study of AV-951 (KRN951), a potent and selective VEGFR-1, -2 and -3 tyrosine kinase inhibitor, in patients with advanced solid tumors. Annual Meeting of the American Association for Cancer Research, April 2008: abstract LB-201

[bib14] Feldman DR, Kondagunta GV, Ronnen EA, Fischer P, Chang R, Baum M, Ginsberg MS, Ishill N, Patil S, Motzer RJ (2007) Phase I trial of bevacizumab plus sunitinib in patients (pts) with metastatic renal cell carcinoma (mRCC). J Clin Oncol. ASCO Annual Meeting Proceedings Part I. Vol 25, No. 18S (20 June Supplement): (Abstract 5099)

[bib15] Ferlay J, Autier P, Boniol M, Heanue M, Colombet M, Boyle P (2007) Estimates of the cancer incidence and mortality in Europe in 2006. Ann Oncol 18: 581–592 17287242 10.1093/annonc/mdl498

[bib16] Fisher RI, Rosenberg SA, Fyfe G (2000) Long-term survival update for high-dose recombinant interleukin-2 in patients with renal cell carcinoma. Cancer J Sci Am 6(Suppl 1): S55–S57 10685660

[bib17] Hainsworth JD, Spigel DR, Burris III HA, Waterhouse D, Clark BL, Whorf R (2010) Phase II trial of bevacizumab and everolimus in patients with advanced renal cell carcinoma. J Clin Oncol 28: 2131–2136 20368560 10.1200/JCO.2009.26.3152

[bib18] Houk BE, Bello CL, Poland B, Rosen LS, Demetri GD, Motzer RJ (2010) Relationship between exposure to sunitinib and efficacy and tolerability endpoints in patients with cancer: results of a pharmacokinetic/pharmacodynamic meta-analysis. Cancer Chemother Pharmacol 66: 357–371 19967539 10.1007/s00280-009-1170-y

[bib19] Huang D, Ding Y, Zhou M, Rini BI, Petillo D, Qian C-N, Kahnoski R, Futreal PA, Furge KA, Teh BT (2010) Interleukin-8 mediates resistance to antiangiogenic agent sunitinib in renal cell carcinoma. Cancer Res 70: 1063–1071 20103651 10.1158/0008-5472.CAN-09-3965 PMC3719378

[bib20] Hudes G, Carducci M, Tomczak P, Dutcher J, Figlin R, Kapoor A, Staroslawska E, Sosman J, McDermott D, Bodrogi I, Kovacevic Z, Lesovoy V, Schmidt-Wolf IG, Barbarash O, Gokmen E, O’Toole T, Lustgarten S, Moore L, Motzer RJ (2007) Temsirolimus, interferon alfa, or both for advanced renal-cell carcinoma. N Engl J Med 356: 2271–2281 17538086 10.1056/NEJMoa066838

[bib21] Krause DS, Van Etten RA (2005) Tyrosine kinases as targets for cancer therapy. N Engl J Med 353: 172–187 16014887 10.1056/NEJMra044389

[bib22] Le Tourneau C, Faivre S, Serova M, Raymond E (2008) mTORC1 inhibitors: is temsirolimus in renal cancer telling us how they really work? Br J Cancer 99(8): 1197–1203 18797463 10.1038/sj.bjc.6604636 PMC2570519

[bib23] McDermott DF, Ghebremichael MS, Signoretti S, Margolin KA, Clark J, Sosman JA, Dutcher JP, Logan T, Figlin RA, Atkins MB, on behalf of the Cytokine Working Group (2010) The high-dose aldesleukin (HD IL-2) ‘SELECT’ trial in patients with metastatic renal cell carcinoma (mRCC). J Clin Oncol 28(15s) (Abstract 4514)

[bib24] McDermott DF, Regan MM, Clark JI, Flaherty LE, Weiss GR, Logan TF, Kirkwood JM, Gordon MS, Sosman JA, Ernstoff MS, Tretter CP, Urba WJ, Smith JW, Margolin KA, Mier JW, Gollob JA, Dutcher JP, Atkins MB (2005) Randomized phase III trial of high-dose interleukin-2 versus subcutaneous interleukin-2 and interferon in patients with metastatic renal cell carcinoma. J Clin Oncol 23: 133–141 15625368 10.1200/JCO.2005.03.206

[bib25] Medina MA, Munoz-Chapuli R, Quesada AR (2007) Challenges of antiangiogenic cancer therapy: trials and errors, and renewed hope. J Cell Mol Med 11: 374–382 17635633 10.1111/j.1582-4934.2007.00056.x PMC3922346

[bib26] Merchan JR, Lui G, Fitch T, Picus J, Qin R, Pitot HC, Maples W, Erlichman C (2007) Phase I/II trial of CCI-779 and bevacizumab in stage IV renal cell carcinoma: phase I safety and activity results. J Clin Oncol 2007: 25. ASCO Annual Meeting Proceedings Part I. Vol 25, No. 18S (20 June Supplement): (Abstract 5034)

[bib27] Motzer RJ, Bander NH, Nanus DM (1996) Renal-cell carcinoma. N Engl J Med 335: 865–875 8778606 10.1056/NEJM199609193351207

[bib28] Motzer RJ, Bukowski RM (2006) Targeted therapy for metastatic renal cell carcinoma. J Clin Oncol 24: 5601–5608 17158546 10.1200/JCO.2006.08.5415

[bib29] Motzer RJ, Escudier B, Oudard S, Hutson TE, Porta C, Bracarda S, Grunwald V, Thompson JA, Figlin RA, Hollaender N, Urbanowitz G, Berg WJ, Kay A, Lebwohl D, Ravaud A (2008) Efficacy of everolimus in advanced renal cell carcinoma: a double-blind, randomised, placebo-controlled phase III trial. Lancet 372: 449–456 18653228 10.1016/S0140-6736(08)61039-9

[bib30] Motzer RJ, Escudier B, Oudard S, Hutson TE, Porta C, Bracarda S, Grunwald V, Thompson JA, Figlin RA, Hollaender N, Kay A, Ravaud A (2010) Phase 3 trial of everolimus for metastatic renal cell carcinoma: final results and analysis of prognostic factors. Cancer 116: 4256–4265 20549832 10.1002/cncr.25219

[bib31] Motzer RJ, Hutson TE, Tomczak P, Dror Michaelson M, Bukowski RM, Oudard S, Negrier S, Szczylik C, Pili R, Bjarnason GA, Garcia-del-Muro X, Sosman JA, Solska E, Wilding G, Thompson JA, Kim ST, Chen I, Huang X, Figlin RA (2009) Overall survival and updated results for sunitinib versus interferon alfa in first-line treatment of patients with metastatic renal cell carcinoma. J Clin Oncol 27: 3584–3590 19487381 10.1200/JCO.2008.20.1293 PMC3646307

[bib32] Motzer RJ, Hutson TE, Tomczak P, Michaelson MD, Bukowski RM, Rixe O, Oudard S, Negrier S, Szczylik C, Kim ST, Chen I, Bycott PW, Baum CM, Figlin RA (2007) Sunitinib versus interferon alfa in metastatic renal-cell carcinoma. N Engl J Med 356: 115–124 17215529 10.1056/NEJMoa065044

[bib33] Mulders P, Hawkins R, Nathan P, de Jong I, Osanto S, Porfiri E, Protheroe A, Mookerjee B, Pike L, Gore ME (2009) Final results of a phase II randomised study of cediranib (RECENTIN™) in patients with advanced renal cell carcinoma (RCC). Eur J Cancer Supplements 7: 21 10.1016/j.ejca.2011.12.022 22285180

[bib34] Oudard S, Beuselinck B, Decoene J, Albers P (2010) Sunitinib for the treatment of metastatic renal cell carcinoma. Cancer Treat Rev; e-pub ahead of print 10.1016/j.ctrv.2010.08.005 20817406

[bib35] Parkin DM, Bray F, Ferlay J, Pisani P (2005) Global cancer statistics, 2002. CA Cancer J Clin 55: 74–108 15761078 10.3322/canjclin.55.2.74

[bib36] Patel PH, Senico PL, Curiel RE, Motzer RJ (2009) Phase I study combining treatment with temsirolimus and sunitinib malate in patients with advanced renal cell carcinoma. Clin Genitourin Cancer 7: 24–27 19213664 10.3816/CGC.2009.n.004 PMC3740755

[bib37] Rini BI, Flaherty K (2008) Clinical effect and future considerations for molecularly-targeted therapy in renal cell carcinoma. Urol Oncol 26: 543–549 18774471 10.1016/j.urolonc.2008.03.012

[bib38] Rini BI, Garcia JA, Cooney MM, Elson P, Tyler A, Beatty K, Bokar J, Mekhail T, Bukowski RM, Budd GT, Triozzi P, Borden E, Ivy P, Chen HX, Dowlati A, Dreicer R (2009a) A phase I study of sunitinib plus bevacizumab in advanced solid tumors. Clin Cancer Res 15: 6277–6283 19773375 10.1158/1078-0432.CCR-09-0717 PMC2756318

[bib39] Rini BI, Garcia JA, Cooney MM, Elson P, Tyler A, Beatty K, Bokar J, Ivy P, Chen HX, Dowlati A, Dreicer R (2010c) Toxicity of sunitinib plus bevacizumab in renal cell carcinoma. J Clin Oncol 28(17): e284–e285 20439632 10.1200/JCO.2009.27.1759

[bib40] Rini BI, Halabi S, Rosenberg J, Stadler WM, Vaena DA, Atkins JN, Picus J, Czaykowski P, Dutcher J, Small EJ (2009b) Bevacizumab plus interferon-alpha versus interferon-alpha monotherapy in patients with metastatic renal cell carcinoma: results of overall survival for CALGB 90206. J Clin Oncol 27. Abstr LBA5019 – Oral presentation

[bib41] Rini BI, Halabi S, Rosenberg JE, Stadler WM, Vaena DA, Archer L, Atkins JN, Picus J, Czaykowski P, Dutcher J, Small EJ (2010b) Phase III trial of bevacizumab plus interferon alfa versus interferon alfa monotherapy in patients with metastatic renal cell carcinoma: final results of CALGB 90206. J Clin Oncol 28: 137–143 10.1200/JCO.2009.26.5561 PMC2860433 20368558

[bib42] Rini BI, Hutson TE, Elson P, Heng DY, Knox JJ, Michaelson D, Choueiri TK, Escudier BJ (2010a) Clinical activity of sunitinib rechallenge in metastatic renal cell carcinoma. American Society for Clinical Oncology – Genitourinary Cancers Symposium. Abstr 319 – Oral presentation

[bib43] Rini BI, Michaelson MD, Rosenberg JE, Bukowski RM, Sosman JA, Stadler WM, Hutson TE, Margolin K, Harmon CS, DePrimo SE, Kim ST, Chen I, George DJ (2008) Antitumor activity and biomarker analysis of sunitinib in patients with bevacizumab-refractory metastatic renal cell carcinoma. J Clin Oncol 26: 3743–3748 18669461 10.1200/JCO.2007.15.5416

[bib44] Rini BI, Wilding G, Hudes G, Stadler W, Kim SJ (2007) Axitinib (AG-013736) in patients with metastatic renal cell cancer (RCC) refractory to sorafenib. J Clin Oncol 25: 242S 10.1200/JCO.2008.21.7034 19652060

[bib45] Rixe O, Bukowski RM, Michaelson MD, Wilding G, Hudes GR, Bolte O, Motzer RJ, Bycott P, Liau KF, Freddo J, Trask PC, Kim S, Rini BI (2007) Axitinib treatment in patients with cytokine-refractory metastatic renal-cell cancer: a phase II study. Lancet Oncol 8: 975–984 17959415 10.1016/S1470-2045(07)70285-1

[bib46] Ryan CW, Goldman BH, Lara Jr PN, Mack PC, Beer TM, Tangen CM, Lemmon D, Pan CX, Drabkin HA, Crawford ED (2007) Sorafenib with interferon alfa-2b as first-line treatment of advanced renal carcinoma: a phase II study of the Southwest Oncology Group. J Clin Oncol 25: 3296–3301 17664477 10.1200/JCO.2007.11.1047

[bib47] Sablin M, Bouaita L, Balleyguier C, Gautier J (2007) Sequential use of sorafenib and sunitinib in renal cancer: retrospective analysis in 90 patients. J Clin Oncol. ASCO Annual Meeting Proceedings Part I. Vol. 25, No. 18S (June 20 Supplement):(Abstract 5038)

[bib48] Schmidinger M, Bellmunt J (2010) Plethora of agents, plethora of targets, plethora of side effects in metastatic renal cell carcinoma. Cancer Treat Rev 36(5): 416–424 20163917 10.1016/j.ctrv.2010.01.003

[bib49] Schmidt C (2008) Resistance revisited: looking back at 10 years of multidrug resistance research. J Natl Cancer Inst 100: 1428–1429 18840811 10.1093/jnci/djn375

[bib50] Sessa C, Guibal A, Del CG, Ruegg C (2008) Biomarkers of angiogenesis for the development of antiangiogenic therapies in oncology: tools or decorations? Nat Clin Pract Oncol 5: 378–391 18560389 10.1038/ncponc1150

[bib51] Sosman JA, Flaherty K, Atkins MB (2006) A phase I/II trial of sorafenib (S) with bevacizumab (B) in metastatic renal cell cancer (mRCC) patients (Pts). J Clin Oncol 24(Suppl. 18S): 128s

[bib52] Sosman JA, Flaherty KT, Atkins MB, McDermott DF, Rothenberg ML, Vermeulen WL, Harlacker K, Hsu A, Wright JJ, Puzanof I (2008) Updated results of phase I trial of sorafenib (S) and bevacizumab (B) in patients with metastatic renal cell cancer (mRCC). Poster presented at the 44th Annual Meeting of the American Society of Clinical Oncology, Chicago, Illinois, 30 May–3 June 2008 (Abstract 5011)

[bib53] Sosman JA, Puzanov I, Atkins MB (2007) Opportunities and obstacles to combination targeted therapy in renal cell cancer. Clin Cancer Res 13: 764s–769s 17255307 10.1158/1078-0432.CCR-06-1975

[bib54] Sternberg CN, Davis ID, Mardiak J, Szczylik C, Lee E, Wagstaff J, Barrios CH, Salman P, Gladkov OA, Kavina A, Zarbá JJ, Chen M, McCann L, Pandite L, Roychowdhury DF, Hawkins RE (2010a) Pazopanib in locally advanced or metastatic renal cell carcinoma: results of a randomized phase III trial. J Clin Oncol 28: 1061–1068 20100962 10.1200/JCO.2009.23.9764

[bib55] Sternberg CN, Hawkins R, Szczylik C, Davis ID, Wagstaff J, McCann L, Chen M, Rubin SD (2010b) Randomized, double-blind phase III study of pazopanib in patients with advanced metastatic renal cell carcinoma (mRCC): final overall survival (OS) results. Ann Oncol 21(Suppl. 8): viii10 Abstract LBA22

[bib56] Tamaskar I, Shaheen P, Wood L (2006) Antitumor effects of sorafenib and sunitinib in patients (pts) with metastatic renal cell carcinoma (mRCC) who had prior therapy with anti-angiogenic agents. J Clin Oncol 24: 4597

[bib57] Tannir N, Wong Y, Kollmannsberger C, Ernstoff MS, Perry DJ, Appleman LJ, Posadas E, Qian J, Ricker JL, Michaelson MD (2009) Phase 2 results of ABT-869 treatment in patients with advanced renal cell cancer (RCC) after sunitinib failure. Eur J Cancer Supplements 7: 425 10.1016/j.ejca.2011.09.002 PMC4167844 22078932

[bib58] Toschi A, Lee E, Gadir N, Ohh M, Foster DA (2008) Differential dependence of hypoxia-inducible factors 1*α* and 2*α* on mTORC1 and mTORC2. J Biol Chem 283: 34495–34499 18945681 10.1074/jbc.C800170200 PMC2596400

[bib59] Whorf RC, Hainsworth JD, Spigel DR, Yardley DA, Burris III HA, Waterhouse DM, Vazquez ER, Greco FA (2008) Phase II study of bevacizumab and everolimus (RAD001) in the treatment of advanced renal cell carcinoma (RCC). Poster presented at the 44th Annual Meeting of the American Society of Clinical Oncology, Chicago, Illinois, 30 May–3 June 2008. http://www.clinicaltrials.gov (2010) http://clinicaltrials.gov/

